# Examining Insulin Resistance and BMI in the Context of Alpha-Klotho and Functional Decline

**DOI:** 10.1093/geroni/igaf122.2879

**Published:** 2025-12-31

**Authors:** Anam Ahmad, Alan Rathbun, Michelle Shardell, Richard Semba, Rita Kalyani, Luigi Ferrucci

**Affiliations:** University of Maryland School of Medicine, Cary, North Carolina, United States; University of Maryland School of Medicine, Baltimore, Maryland, United States; University of Maryland School of Medicine, Baltimore, Maryland, United States; Johns Hopkins University, Baltimore, Maryland, United States; Johns Hopkins University School of Medicine, Baltimore, Maryland, United States; NIA, Bethesda, Maryland, United States

## Abstract

Alpha-klotho, a longevity-associated protein, has been linked to aging-related physical and cognitive decline. Research implies that alpha-klotho is associated with insulin resistance (IR), and with cognitive and physical decline. Body mass index (BMI) is also a risk factor for IR and both functional declines. However, it is unknown whether IR mediates the relationship between alpha-klotho and dual cognitive-physical decline, or if this relationship differs by BMI. Therefore, we assessed mediation with stratification by BMI. Older adults (n = 461, age ≥65 years) from the Invecchiare in Chianti study were phenotypically categorized based on cognitive (Mini-Mental State Examination) and physical (4-meter gait speed) using group-based trajectory analysis with data up to 15 years of follow-up. Overall, n = 149 (32.3%) participants were categorized as experiencing dual cognitive-physical decline. IR was operationalized using the homeostasis model for insulin resistance (HOMA-IR). Mediation models were implemented to estimate total, natural direct, and natural indirect effects (TE, NDE, NIE, respectively) of alpha-klotho on dual cognitive-physical decline. Adjusted odds ratios (ORs) of dual cognitive-physical decline per doubling of log-transformed alpha-klotho for TE (OR = 0.45, 95% Confidence Interval[CI]= 0.11-1.48), NDE (OR = 0.44, 95%CI= 0.12-1.38) and NIE (OR = 1.01, 95%CI=0.70-1.28) among n = 225 overweight (BMI=25-29.9 kg/m2) participants indicated negligible (-0.5%) mediation by HOMA-IR in the relation between alpha-klotho and dual cognitive-physical decline. No significant effects were found overall or among the obese (n = 68, BMI > =30 kg/m2) or normal/underweight (n = 168, BMI< 25 kg/m2) strata. Further research is needed to better understand potential mechanisms linking alpha-klotho and insulin resistance to cognitive and physical performance over time in older adults.

